# Quercetin-crosslinked chitosan nanoparticles: a potential treatment for allergic rhinitis

**DOI:** 10.1038/s41598-024-54501-2

**Published:** 2024-02-18

**Authors:** Dehong Mu, Li Zhou, Lingyu Shi, Ting Liu, Ying Guo, Hao Chen, Hongping Luo, Junhao Ma, Hui Zhang, Peizheng Xiong, Li Tian

**Affiliations:** 1 Department of Otorhinolaryngology, Hospital of Chengdu University of Traditional Chinese Medicine, Chengdu, 610072 People’s Republic of China; 2 Department of Nanchong Vocational College of Science and Technology, Nanchong, 637200 People’s Republic of China; 3 Department of Clinical Medicine School of Chengdu, University of Traditional Chinese Medicine, Chengdu, 610075 People’s Republic of China

**Keywords:** Materials science, Nanoparticles, Drug development

## Abstract

Allergic rhinitis (AR) remains a major health problem worldwide. Compared with traditional oral drugs, nasal administration avoids first-pass metabolism and achieve faster and more effective efficacy. In this study, we used the ion crosslinking method to prepare quercetin–chitosan nasal adaptive nanomedicine (QCS) delivery system and evaluated in the treatment of allergic rhinitis mice models. The obtained positively charged nanoparticles with a particle size of 229.2 ± 0.2 nm have excellent characteristics in encapsulation efficiency (79.604%), drug loading rate (14.068%), drug release (673.068 μg) and stability(> 7 days). Excitingly, QCS treatment significantly reduced the number of sneezing and nasal rubbing events in AR mice, while reducing the levels of inflammatory factors such as immunoglobulin E (IgE), interleukin (IL)-17, tumor necrosis factor (TNF)-α, and (IL)-6 to alleviate AR symptoms. Hematoxylin–eosin (HE) staining also showed the damaged nasal mucosa was improved. These experimental results suggest that QCS can effectively suppress allergic inflammation in a mouse model and hold promise as a therapeutic option for allergic rhinitis.

## Introduction

**Scheme 1:**
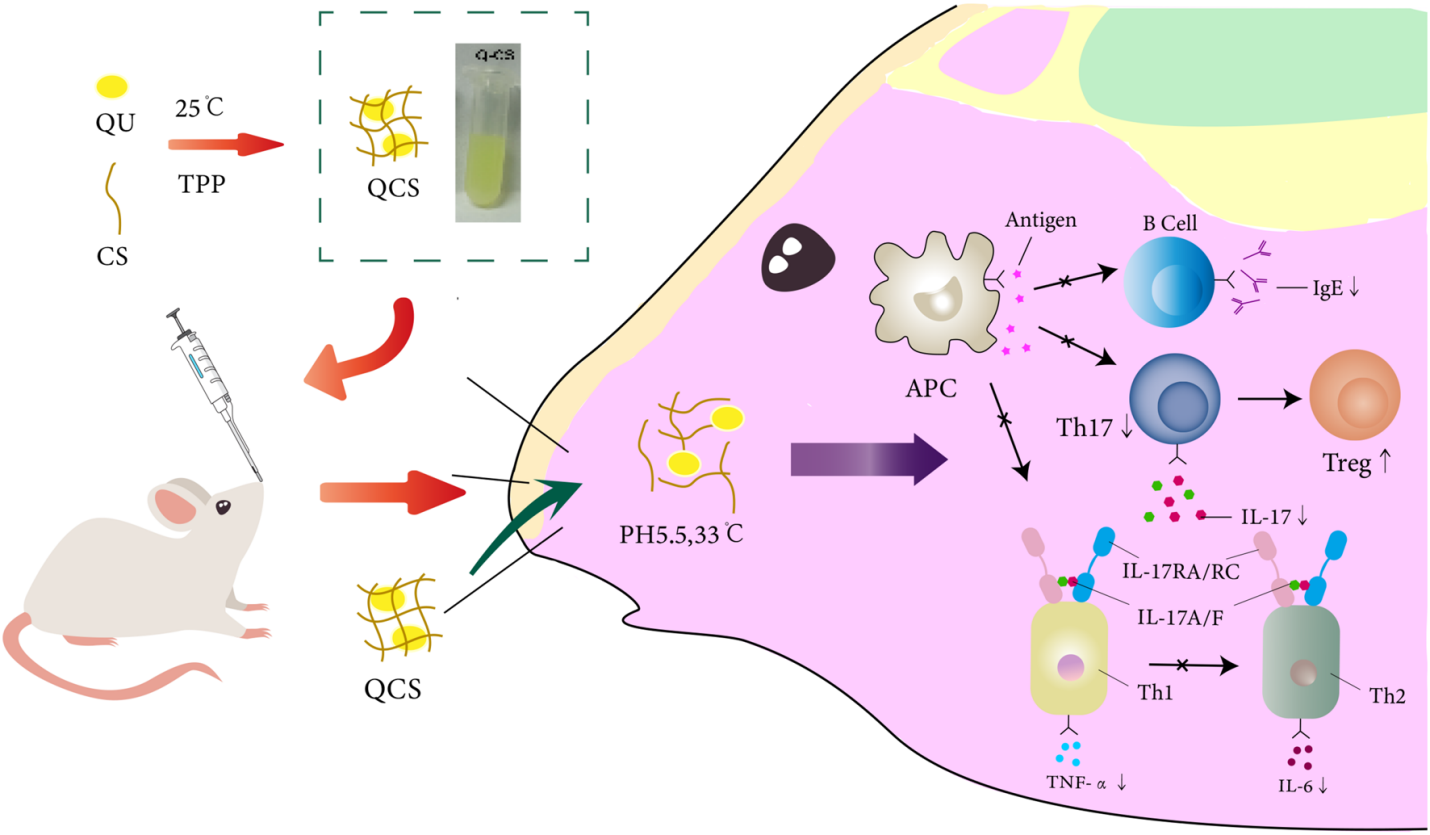
Schematic representation of the therapeutic mechanism of QCS.

Allergic rhinitis (AR) is a chronic inflammatory disease of the nasal mucosa characterized by symptoms such as nasal itching, intermittent sneezing, profuse watery rhinorrhea, and nasal congestion. These are mainly mediated by immunoglobulin E (IgE) after susceptible individuals have been exposed to allergens^1^. AR has the characteristics of recurrent episodes and difficulty in achieving a cure. Recent epidemiological surveys have shown an overall increase in the prevalence of AR both in Chinaand internationally^2^. Owing to the influence of environmental pollution and other factors, the efficacy of AR treatment is not satisfactory. Therefore, the treatment of AR has always been a popular topic of research. In the pathogenesis of AR, an allergen comes into contact with the human body and is taken up by antigen-presenting cells (APCs), which then present it to T helper (Th) cells by binding with major histocompatibility complex molecules, which leads to immune imbalance. Common clinical treatment options for AR include antihistamines, corticosteroids, and oral leukotriene inhibitors. However, continuous treatment can cause varying degrees of side effects. In addition, AR involves diverse allergens, affects a younger population, and has a long disease course and a propensity to relapse, which further complicate the treatment of AR. Nasal sprays are common treatments for rhinitis that can alleviate rhinitis symptoms by being directly sprayed into the nasal cavity. They act directly at the site of inflammation and provide rapid relief. Moreover, they have relatively few side effects in comparison with oral medications and injection treatments, which makes them safer. Nasal sprays are simple and convenient to carry and do not interfere with the patient's normal life and work. However, since the nasal spray has been produced for decades, there are only hormones, and long-term use may still increase drug resistance and cause problems such as drug dependence. Although clinical practice has been devoted to improving treatment methods, the development of new drugs that combine low toxicity, minimal side effects, and high efficacy represents an innovative strategy. For example, nanoparticle formulations are produced by the application of nanocarrier technology to convert active pharmaceutical ingredients into nanoparticles (Scheme 1).

These nanoparticles, which have nanoscale dimensions, can be combined with drugs via electrostatic adsorption or covalent or noncovalent bonds or even encapsulated within the carrier^3^. In comparison with other dosage forms, nanoparticle formulations exhibit significant advantages in terms of improving the bioavailability of drugs, enhancing stability, reducing drug toxicity and side effects, prolonging drug circulation times, providing diverse administration routes, and facilitating targeted drug delivery. Chitosan (CS) is a biodegradable, nonantigenic, nontoxic, and biocompatible natural polymer that has been shown to display various beneficial effects on health, including potent antioxidant and antimicrobial activities^4^. CS and its derivatives exhibit various promising biological activities, which make them suitable as antimicrobial agents. CS, which is a cationic polymer, possesses unique properties that allow its safe utilization in wastewater treatment, pharmacology, and biomedical applications. Owing to their distinctive physical and chemical characteristics, CS and its derivatives are considered excellent sources for the production of safe and effective drug delivery systems. Another significant attribute of CS is its strong adhesiveness, which is attributed to electrostatic interactions between the positively charged amino groups on the polymer chains and the negatively charged residues of mucins rich in sialic acid and sulfonic acid groups in the nasal cavity. Furthermore, the acidic microenvironment (pH of 5.5, lysozyme concentration of approximately 30 μg/mL) within the nasal cavity stimulates the responses of CS, which enable the release of loaded drugs. Therefore, CS can be utilized for the delivery of nasal mucosal antiallergens, prolonging drug residence times in the nasal cavity, enhancing bioavailability, and minimizing irritation caused by encapsulated payloads.

Quercetin (QU), which is a type of bioflavonoid, is found in over 100 traditional Chinese medicines (such as *Astragalus membranaceus*, *Platycladus orientalis* leaves, *Alpinia officinarum*, *Lonicera japonica* flowers, Sangbaipi, *Panax notoginseng*, and *Ginkgo biloba*)^5–7^. It exhibits significant physiological functions, including scavenging free radicals, and has antioxidant, antiaging, immunomodulatory, and antitumor properties, and it thereby prevents chronic diseases^8^. Inflammatory responses of the body occur during tissue injury, bacterial infections, and prolonged oxidative stress, which may lead to various chronic diseases, such as cardiovascular disorders, neurodegenerative diseases, and obesity^9^. During oxidative reactions, the hydroxyl groups in flavonoids such as QU and myricetin can donate hydrogen atoms, which react with free radicals to form quinone-type free radical intermediates. These tend to be stabilized by aromatic ring conjugation, which thus interrupts the propagation of oxidative chain reactions and prevents peroxidation processes^10^.

QU and other flavonoids display strong anti-inflammatory activity, which is mainly achieved via the following mechanisms: (1) inhibition of proinflammatory protein kinases, cyclooxygenase-2, and transcription factors such as nuclear factor-κB (NF-κB), GATA-3, and signal transducer and activator of transcription-6 (STAT-6); (2) scavenging of reactive oxygen species, which reduces their accumulation in the body; and (3) modulation of immune cell activity, such as inhibition of cell activation, maturation, signal transduction, and secretion processes, which thereby suppresses inflammatory reactions^10,11^. Flavonoids, including QU, rutin, cyanidin, naringenin, and hesperidin, as well as catechins and anthocyanins, have been demonstrated to possess anti-inflammatory properties in in vitro and in vivo models, as well as clinical studies^12,13^, by regulating protein kinases via the inhibition of transcription factors such as NF-κB^14^. In addition, QU can modulate the transcription factors of CD4^+^ T helper 2 (Th2) cells, such as GATA-3, as well as STAT-6, and thereby inhibit inflammatory responses^15,16^.

Despite the therapeutic effects of QU on numerous diseases, its low oral bioavailability has restricted its application in the field of medicine. This is due to factors such as poor water solubility, limited permeability through cell membranes, instability in physiological media (stomach and intestines), a short biological half-life, and extensive first-pass metabolism in the liver before it reaches the systemic circulation. Therefore, it is imperative to seek solutions for overcoming the poor bioavailability of QU and enhancing its biological activity. One possible approach is the utilization of topical nanodrug delivery, wherein QU is loaded onto a CS nanoplatform for intranasal administration. In the slightly acidic environment of the nasal cavity, this system stimulates the responses of CS, which lead to the release of QU. Moreover, CS facilitates drug dissolution and thus improves drug bioavailability. By mutually complementing each other, this synergistic effect of the QU-loaded CS nanodrug platform achieves better therapeutic outcomes in AR. The subsequent synthesis of QU@CS (QCS) and preliminary evaluation of its effects on living organisms, as well as observations of its therapeutic efficacy, are described below.

## Materials and methods: QCS preparation

### Materials

CS (viscosity of < 200 mPa s, deacetylation degree of 97.8%) was purchased from Pudong Shanghai McLean Company (C804726 100 g). Sodium tripolyphosphate (TPP) was purchased from Pudong, Shanghai McLean Company (S817361 500 g). QU (content of ≥ 95%) was purchased from Sigma Reagent Company (Massachusetts,USA) (Q4951 10 g). Acetic acid solution was purchased from Pudong Shanghai Aladdin Company. Egg albumin, aluminum hydroxide, 0.9% physiological saline, and 1.5% isoflurane were all purchased from Pudong Shanghai Hengyuan Biotechnology Co., Ltd.

### Synthesis and analysis of QCS

#### Synthetic method

Firstly, CS was dissolved in acetic acid solution to lower its pH. Then, various doses of an ethanolic solution of QU were added with continuous stirring. Next, TPP solution was introduced to enhance particle stability and promote dispersion of the suspension. Finally, the suspension was centrifuged and the precipitate was collected, followed by freeze-drying for preservation.

#### Particle size and zeta potential analysis

The characteristics of QCS were investigated using hydrated particle size and zeta potential tests. The hydrated particle size refers to the average distance between water molecules and ions in the hydrated compound. In general, a smaller hydrated particle size indicates that the hydrated compound has a more compact structure. Zeta potential, which is also known as surface potential, represents the amount of charge carried by particles on their surfaces and reflects the stability of the particle system. A sample of approximately 0.5–1 mL QCS was added to the sample vessel of a Zetasizer Nano particle size and zeta potential analyzer. The hydrated particle size and zeta potential were measured at 25 °C. The sample concentration used was 1 mg/mL. Three measurements were taken for each indicator to determine the particle size and zeta potential of QCS.

#### Temporal stability study

To assess the temporal stability of the prepared QCS, the hydrated particle size and polydispersity index (PDI) were measured using a Malvern particle size analyzer on day 1, day 4, and day 7. The QCS samples were dispersed in an aqueous solution for analysis.

#### Fourier transform infrared spectroscopy characterization

The Fourier transform infrared (FT-IR) spectra of CS, QU, and QCS were obtained using an FT-IR spectrometer (PerkinElmer, Massachusetts, USA). Infrared radiation passing through a sample is partially absorbed and partially transmitted. The signal generated in the detector is presented as a spectrum that represents the molecular "fingerprint" of the sample. For different chemical structures (molecules) different spectral fingerprints are produced, which demonstrates the value of FT-IR spectroscopy. For analysis, 20 mg samples of CS, QU, and QCS powders were mixed with dried KBr powder, ground, and pressed into pellets. Their FT-IR spectra were acquired to infer their chemical structures on the basis of their characteristic absorption peaks.

#### Standard curve calculation for QU

Solutions of QU in anhydrous ethanol with concentrations of 10, 25, 50, 100, and 200 μg/mL were taken. Their absorption peaks at 370 nm were measured using UV spectroscopy, and a standard curve was then created.

#### Determination of encapsulation efficiency and drug loading

The in vitro release performance of the drug delivery system was evaluated using the dialysis bag method. A 10 mg sample of QCS powder was dissolved in 3 mL phosphate-buffered saline (PBS) (pH 7.4), and the solution was transferred to a dialysis membrane bag (molecular weight cutoff of 12 kDa). The bag was immersed in a beaker containing 100 mL PBS (simulating the nasal pH of 6.5), which was stirred constantly at 37 °C (100 rpm). At fixed time intervals (0, 2, 6, 12, 24, 36, and 48 h), 3 mL samples were collected from the beaker and replaced with 3 mL fresh PBS solution. The absorbance of the samples at 370 nm was measured, and the amount of QU released was determined using the QU standard curve. Each experiment was repeated three times, and the cumulative release rate of QU was calculated and analyzed. The drug loading and encapsulation efficiency were calculated using the following formulae:

1 $$\mathrm{Drug \,loading }= (\mathrm{Mass \,of \,loaded \,drug}/\mathrm{Total \,mass \,of \,carrier \,including \,the \,carrier \,and \,the \,loaded \,drug}) \times 100\mathrm{\%}$$

2 $$\mathrm{Encapsulation \,efficiency }= (\mathrm{Mass \,of \,encapsulated \,drug}/\mathrm{Total \,mass \,of \,drug \,added}) \times 100\%$$

#### Data analysis

Origin 8 software was used for statistical analysis. All data are presented as the mean ± standard deviation. Statistical significance was determined when the obtained p value was less than 0.05.

### Ethical approval

All experimental procedures on animals were approved by the Institutional Animal Care and Use Committee (IACUC:2023DL-016) of the Affiliated Hospital of Chengdu University of Traditional Chinese Medicine in Chengdu, SiChuan, China.

## Effectiveness study of QCS in mouse model of ovalbumin-induced AR

### Modeling, grouping, administration, and sampling methods

#### Establishment of mouse model of AR

Twenty male specific-pathogen-free BALB/c mice with ages of 6 weeks and weights ranging from 18 to 20 g were purchased from an external source and acclimated for 1 week. On the basis of their weight, the mice were randomly divided into four groups, namely, a control group, an AR model group, a QU group, and a QCS group, with five mice per group. A suspension was prepared by mixing 0.5 mg ovalbumin, 5 mg Al(OH)_3_ powder, and 1 mL saline and was administered by intraperitoneal injection on days 1, 5, 10, and 14, i.e., a total of four injections, to induce sensitization. From day 15 to day 28 all groups were intranasally immunized with 20 μL saline containing ovalbumin at a concentration of 25 mg/mL twice daily for a total of 14 times to induce AR. Within 30 min after the final intranasal sensitization, the mice were observed for nose scratching, sneezing, nasal discharge, and foraging behavior. The scoring system used for the observations was as follows: (1) nasal itching: mild scratching one or two times scored 1 point, frequent scratching of the nose scored 2 points, and scratching all over the body scored 3 points; (2) sneezing: one to three sneezes scored 1 point, four to ten sneezes scored 2 points, and more than 11 sneezes scored 3 points; and (3) clear nasal discharge: discharge reaching the nostrils scored 1 point, discharge beyond the front nostrils scored 2 points, and discharge covering the whole face scored 3 points. A total score of greater than 5 indicated successful modeling of AR.

Institutional Review Board Statement: All experimental procedures on animals were approved by the Institutional Animal Care and Use Committee (IACUC:2023DL-016) of the Affiliated Hospital of Chengdu University of Traditional Chinese Medicine in Chengdu, SiChuan, China. We guarantee that all methods are reported in accordance with the ARIVE guidelines, all methods were carried out in accordance with relevant guidelines and regulations.

#### Drug treatment method

Using a microsyringe, each group received 5 μL of the corresponding drug in each nostril twice daily. The control group and model group received 5 μL vehicle (saline) in each nostril twice daily.

#### Animal handling

Two hours after the final treatment, the mice were anesthetized with isoflurane, and blood samples were collected for an enzyme-linked immunosorbent assay (ELISA) to detect specific IgE. Nasal lavage fluid was collected for an ELISA to detect interleukin (IL)-17, tumor necrosis factor (TNF)-α, and IL-6. Nasal mucosal tissue was stained with hematoxylin and eosin (H&E).

#### Toxicity level assessment

The weight of the mice was measured, and, after euthanasia, the spleen and liver weights were recorded. By comparing the body weight and organ weights between the groups, the effects of the drugs on changes in body weight and organ weight in mice were assessed, and the toxicity levels of the drugs were preliminarily determined.

#### Measurement of parameters of inflammation

The levels of different markers of inflammation were determined before and after treatment to evaluate the efficacy of QCS. These markers included: (1) serum-specific IgE, which can indicate immediate-type reactions such as AR; (2) IL-17, because IL-17 signaling can control inflammation by regulating the expression of proinflammatory genes in most nonhematopoietic cells17; (3) TNF-α, which is also known as cachectin and is a pleiotropic cytokine that plays a central role in inflammation, apoptosis, and immune system development. TNF-α is also involved in the T helper 17 (Th17) signaling pathway, where it interacts with dendritic cells and tissue cells to produce IL-12 and IL-23. IL-23 then participates in the initial differentiation of T cells into Th17 cells, which in turn secrete IL-17A, IL-17F, IL-6, IL-22, IL-26, and TNF-α ^18^; and (4) IL-6, because IL-6 produced by APCs can regulate the differentiation of CD4^+^ T cells into effector T helper 1 (Th1) or Th2 cells. The presence of IL-6 shifts the Th1/Th2 balance toward Th2 via two independent pathways: (1) promoting the production of IL-4 and differentiation of Th2 cells; and (2) inhibiting the production of interferon (IFN)-γ and differentiation of Th1 cells ^19^.

### Statistical analysis

Data were coded and entered using GraphPad Prism version 8 software for statistical analysis. Differences between groups were evaluated using the chi-squared test (for qualitative variables), the t-test for independent samples, and analysis of variance. The post hoc Bonferroni test was employed for pairwise comparison of normally distributed quantitative variables. Differences were considered to be statistically significant when the p value was equal to or less than 0.05.

## Results

### Nanoparticle preparation

#### Study of loading capacity of CS for QU

The study investigated the loading capacity of CS for QU at different feed ratios (pure CS, 8:1, 6:1, 4:1, and 2:1) to determine the optimal feed ratio. The absorbance, QU concentration, QU volume, drug loading mass, particle mass, encapsulation efficiency, and drug loading capacity were compared among the five groups (Table 1). The QCS formulation with a feed ratio of 6:1 exhibited an absorbance of 0.384, a QU concentration of approximately 44.9%, a QU volume of 15%, a drug loading mass of 673.068 μg, an encapsulation efficiency of 79.6%, and a drug loading capacity of 14%. Although the formulation with a feed ratio of 8:1 exhibited a higher encapsulation efficiency of 84.4%, the drug loading capacity was only 11%, and the drug loading mass was significantly lower at 388.447 μg. When the feed ratio was 4:1, the encapsulation efficiency decreased, and the QU concentration became excessively high. Consequently, the feed ratio of 6:1 was determined to be the optimal choice, which provided a drug loading capacity of approximately 14%.

**Table 1:** Test data for quercetin@chitosan (QCS) formulations with different feed ratios.

Group number	1	2	3	4	5
Feed ratio	0	8:1	6:1	4:1	2:1
Drug loading mass(μg)	0	388.447	673.068	1440.251	3654.033
Chitosan mass(μg)	20,000.000	20,000.000	20,000.000	20,000.000	20,000.000
EE (%)	0	84.462	79.604	71.195	63.460
DL (%)	0	11.483	14.068	18.311	29.306

EE = encapsulation efficiency; DL = drug loading capacity.

#### Particle size and zeta potential determination

**Figure 1:**
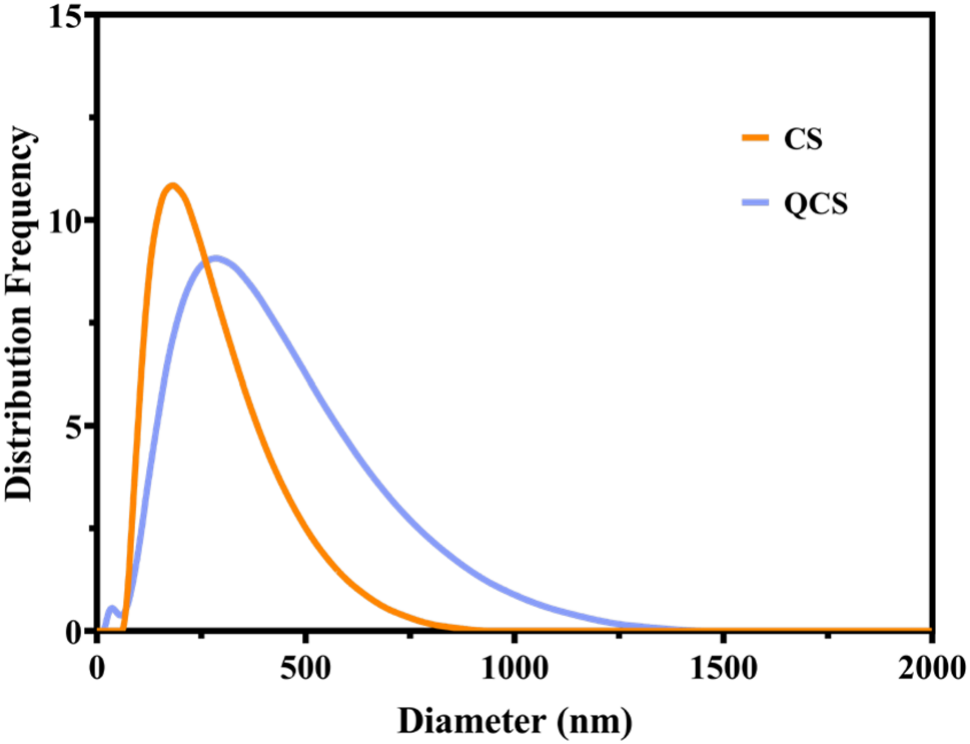
Distribution frequency of chitosan (CS) and QCS.

**Figure 2:**
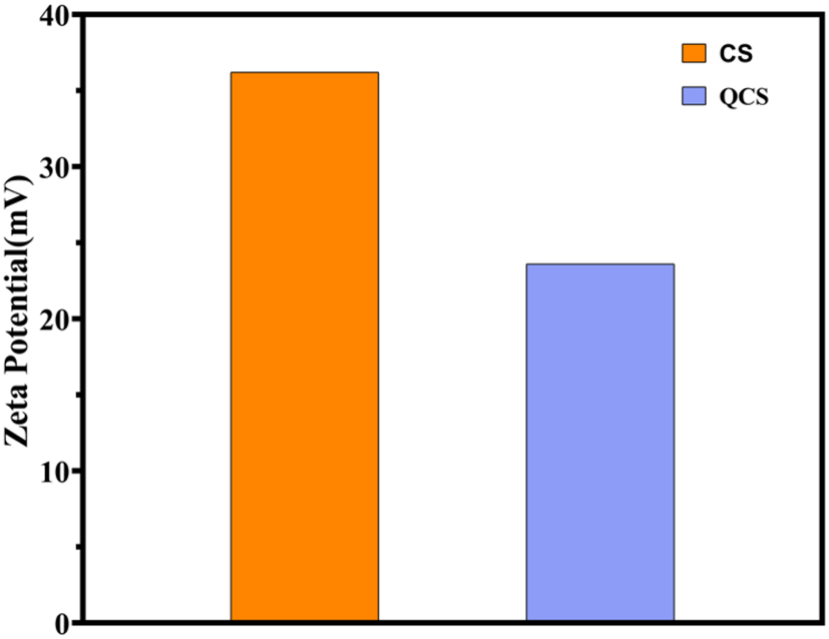
Zeta potential of CS and QCS.

The test results indicated that the hydrated particle size of the prepared nanoparticles was 229.2 ± 0.2 nm, with a uniform particle size distribution (PDI of 0.363) and a zeta potential of 23.6 ± 0.2 mV, which demonstrated high stability. The particle size of the CS raw material was 188.9 ± 0.2 nm, with a PDI of 0.229 and a zeta potential of 36.2 ± 0.2 mV. In comparison with the CS raw material, QCS exhibited a higher particle distribution frequency in the range of 250–1500 nm (Fig. 1) and a lower zeta potential, which indicated increased aggregation (Fig. 2).

#### Temporal stability of QCS

**Figure 3:**
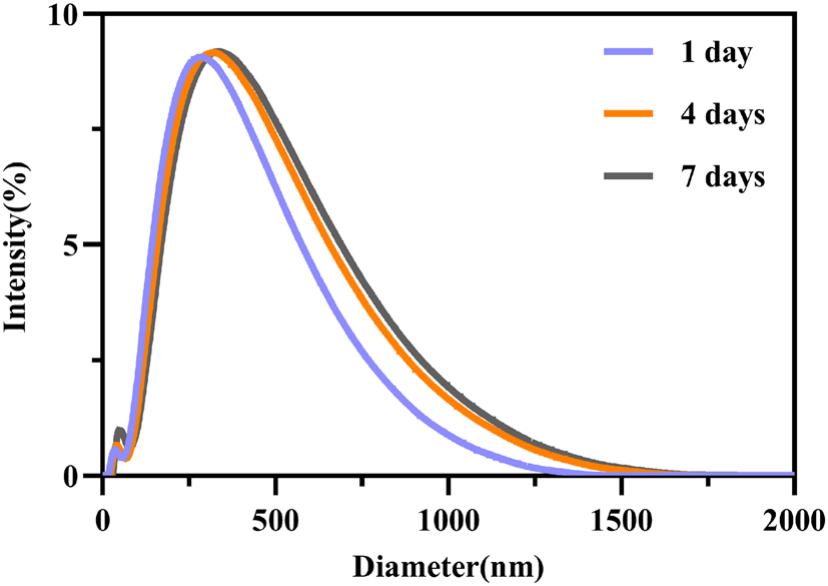
Comparison of distribution frequency of QCS at different time points.

The prepared QCS was dispersed in an aqueous solution, and the hydrated particle size and PDI were measured using a Malvern particle size analyzer on days 1, 4, and 7 to observe its temporal stability (Fig. 3). The results showed that on day 4 the QCS particle size was 274.4 nm with a PDI of 0.315, whereas on day 7 the particle size was 276.4 nm with a PDI of 0.362, which indicated no significant difference (p > 0.05).

#### Infrared spectroscopy study

**Figure 4:**
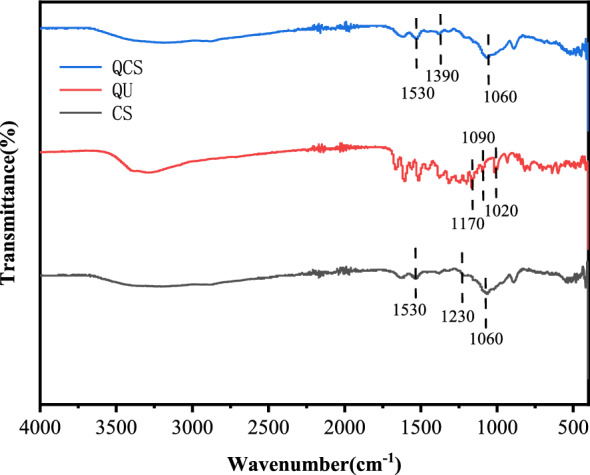
Comparison of Fourier transform infrared spectra of QCS, quercetin (QU), and CS.

A comparison of the FT-IR spectra of 20 mg samples of CS, QU, and QCS powders is shown below (Fig. 4).

On the basis of the peaks in the FT-IR spectrum of CS, the presence of functional groups and their vibrational modes can be inferred as follows: (1) the peak at 549 cm^−1^ may result from the vibrations of furan rings or carboxyl functional groups in CS. The vibrational frequency of furan rings typically falls within the range of 500–600 cm^−1^. (2) The peak at 1060 cm^−1^ may be attributed to the stretching vibrations of C–O bonds in the sugar rings in CS. The vibrational frequency of C–O bonds in sugar rings is commonly observed in the range of 1050–1080 cm^−1^. (3) The peak at 1230 cm^−1^ may correspond to the stretching vibrations of hydroxyl groups in CS. The vibrational frequency of hydroxyl groups in CS usually occurs within the range of 1200–1300 cm^−1^. (4) The peak at 1530 cm^−1^ might be associated with the vibrations of carboxyl functional groups in CS. The stretching vibrational frequency of carboxyl groups typically falls within the range of 1500–1600 cm^−1^.

According to the peak positions in the infrared spectrum of QU, the presence and vibrational modes of certain functional groups can be inferred as follows: (1) the peak at 1020 cm^−1^ may correspond to the stretching vibrations of C–C bonds in the benzene rings in QU. The stretching vibrations of C–C bonds in benzene rings usually occur in the range of 1000–1100 cm^−1^. (2) The peak at 1090 cm^−1^ may be attributed to the stretching vibrations of C–O bonds on the benzene rings in QU. The stretching vibrations of C–O bonds on benzene rings commonly appear in the range of 1000–1250 cm^−1^. (3) The peak at 1170 cm^−1^ could correspond to the stretching vibrations of the C–O–C linkage in QU. The stretching vibrations of C–O–C linkages on benzene rings are typically observed in the range of 1100–1250 cm^−1^.

In the case of QCS, peaks are observed at 890 cm^−1^, 1060 cm^−1^, 1390 cm^−1^, 1530 cm^−1^, and 1610 cm^−1^ in the infrared spectrum. (1) The peak at 890 cm^−1^ is probably associated with the stretching vibrations of C–O–C linkages in CS and indicates the presence of CS. (2) The peak at 1060 cm^−1^ usually corresponds to the vibrations of C–O bonds on aromatic skeletons and suggests the presence of C–O bonds on the aromatic skeleton of QU. (3) The peak at 1530 cm^−1^ generally corresponds to the vibrations of C=C bonds in aromatic skeletons and suggests the presence of vibrations of C=C bonds in the aromatic skeleton of QU. (4) The peak at 1610 cm^−1^ is typically associated with aromatic ketone structures and indicates the presence of stretching vibrations of the C=O bond in the aromatic ketone structure of QU. The presence of characteristic absorption bands of QU and CS at their respective positions without any disappearances suggests that there was a close connection between QU and CS in QCS, and no interactions between them were observed.

#### QU standard curve and drug release curve

**Figure 5:**
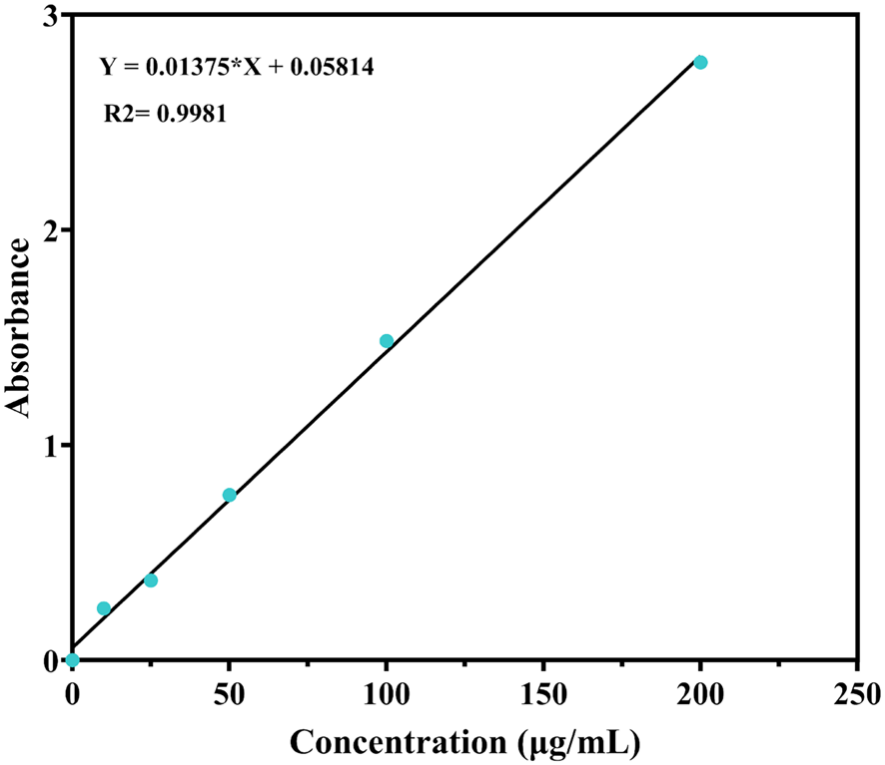
QU standard curve.

**Figure 6:**
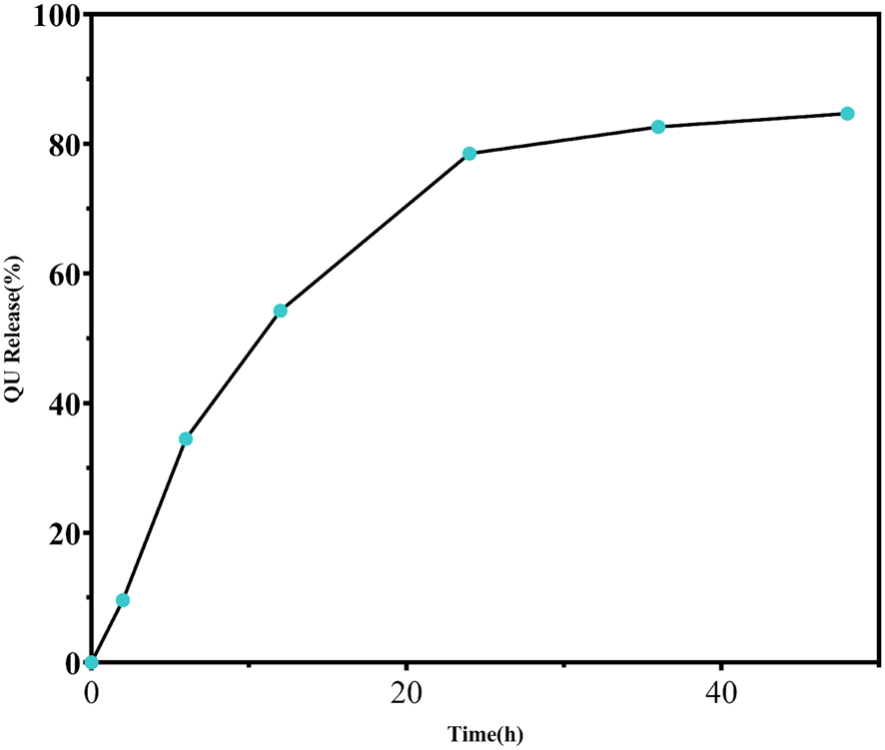
QU release curve.

Concentrated solutions of QU in anhydrous ethanol with concentrations of 10, 25, 50, 100, and 200 μg/mL were prepared, and their absorbances at 370 nm were measured to generate a standard curve with the following regression equation: Y = 0.01375X + 0.05814 (Fig. 5). Subsequently, the rate of release of QU from QCS powder was determined using the dialysis bag method, which resulted in another curve (Fig. 6). It can be observed that the absorbance of QU in the anhydrous ethanolic solution increased linearly with an increase in concentration. The efficiency of the release of QU from QCS in the simulated nasal cavity environment gradually decreased with time. QU exhibited rapid release within 12 h, relatively high overall release within 24 h, and a final release rate of approximately 90%.

### Animal experiment results

#### Toxicity level testing

After the drugs were administered for 14 days, the mice were weighed and sacrificed to measure the weights of their liver and kidneys. The data collected are summarized in Table 2. There was a significant difference (*p* < 0.05) in body weight and liver weight between the model group and the control group, which was possibly due to the suboptimal health condition of the mice in the model group. However, no significant differences in body weight were observed among the other groups in comparison with the control group, which suggested that the drugs had no apparent toxic effect on the mice.

**Table 2:** Comparison of body weight and organ weights among the different groups.

Group	Body weight (g)	Liver weight (g)	Kidney weight (single) (g)
Control group	30.27 ± 1.164	1.74 ± 0.152	0.521 ± 0.126
Model group	22.82 ± 1.738^#^	0.92 ± 0.147^#^	0.366 ± 0.094
QU group	28.42 ± 1.677	1.22 ± 0.196	0.475 ± 0.106
QCS group	27.42 ± 1.194	1.29 ± 0.153	0.465 ± 0.064

The values were compared with those of the control group, with statistical significance defined as ^#^*p* < .05.

#### Symptoms of AR in mice

The symptoms of AR in the mice were evaluated before and after treatment with the different drugs, as shown in Table 3. These symptoms included nasal itching, nasal discharge, and sneezing, which gradually increased during the induction process. Before treatment (day 1), the total symptom scores ranged from 6 to 8. After treatment for 14 days, there was no significant difference in symptom scores between the model group and the QU group (*p* > 0.05). However, treatment with the QCS formulation significantly reduced the symptoms and thus yielded a total score of 1 (*p* < 0.01).

**Table 3:** Comparison of the symptom scores of mice in the different groups.

	Control group		Model group		QU group		QCS group	
	Day 1	Day 14	Day 1	Day 14	Day 1	Day 14	Day 1	Day 14
Nasal irritation	0	0	3	3	2	2	3	1
Sneezing	0	0	2	2	2	1	2	0
Nasal secretion	0	0	3	2	2	1	2	0
Total score	0	0	8	7	6	4	7	1
*p* value	–		> 0.05		> 0.05		0.01	

#### Expression of related factors in mice

The expression levels of IgE, IL-17, TNF-α, and IL-6 in the nasal mucosa of mice were determined after the induction of AR and treatment with the different formulations, as shown in Table 4.

**Table 4:** Immunoglobulin E (IgE) and cytokine concentrations in nasal lavage fluid from mice in each group (mean ± SD).

Group	Sample size (n)	IgE (μg/mL)	IL-17 (pg/mL)	TNF-α (pg/mL)	IL-6 (pg/mL)
Control group	5	4.485 ± 0.801	2.505 ± 0.920	40.849 ± 4.816	5.498 ± 4.287
Model group	5	7.658 ± 1.418**	4.412 ± 1.572**	59.382 ± 7.493**	14.866 ± 3.486**
QU group	5	6.438 ± 1.201^##^	3.926 ± 0.957*	51.649 ± 6.467^#^	11.154 ± 2.162^#^
QCS group	5	5.532 ± 1.151^##&^	2.780 ± 0.583^#&^	40.901 ± 7.562^##&^	7.810 ± 2.960^#&^

*p < .05, **p < .01 in comparison with the control group; ^#^*p* < .05, ^##^*p* < .01 in comparison with the model group; ^&^*p* < .05 in comparison with the QU group. IL = interleukin; TNF = tumor necrosis factor.

In comparison with the control group, the model group had significantly higher concentrations of IgE, IL-17, TNF-α, and IL-6 in nasal lavage fluid (*p* < 0.01), which indicated successful induction of the AR model. The QU group displayed significant increases in these indicators in comparison with the control group (*p* < 0.05), which suggested that QU alone had limited efficacy. In contrast, the QCS group did not exhibit statistically significant differences in the concentrations of these cytokines in comparison with the control group. Considering the alleviation of late-stage symptoms in mice, the efficacy of QCS in treating AR was demonstrated.

In comparison with the model group, the QCS group and the QU group had significantly lower IgE concentrations in nasal lavage fluid (*p* < 0.01). The QCS group had a lower IL-17 concentration in nasal lavage fluid (*p* < 0.05), but the difference in IL-17 concentration in nasal lavage fluid from the QU group was not statistically significant. In comparison with the model group, the QCS group had a significantly lower TNF-α concentration in nasal lavage fluid (*p* < 0.01), and the QU group also had a lower TNF-α concentration in nasal lavage fluid, with a statistically significant difference (*p* < 0.05). In comparison with the model group, the QCS group had a lower IL-6 concentration in the nasal mucosa (*p* < 0.05), while the difference in IL-6 concentration in the nasal mucosa of the QU group was also statistically significant. These findings indicate that QU alone had a slight effect on the symptoms, but its efficacy was not significant.

In comparison with the QU group, the QCS group had significantly lower concentrations of all the indicators (*p* < 0.05), which demonstrated that the QCS nanoplatform had an enhanced therapeutic effect.

**Figure 7:**
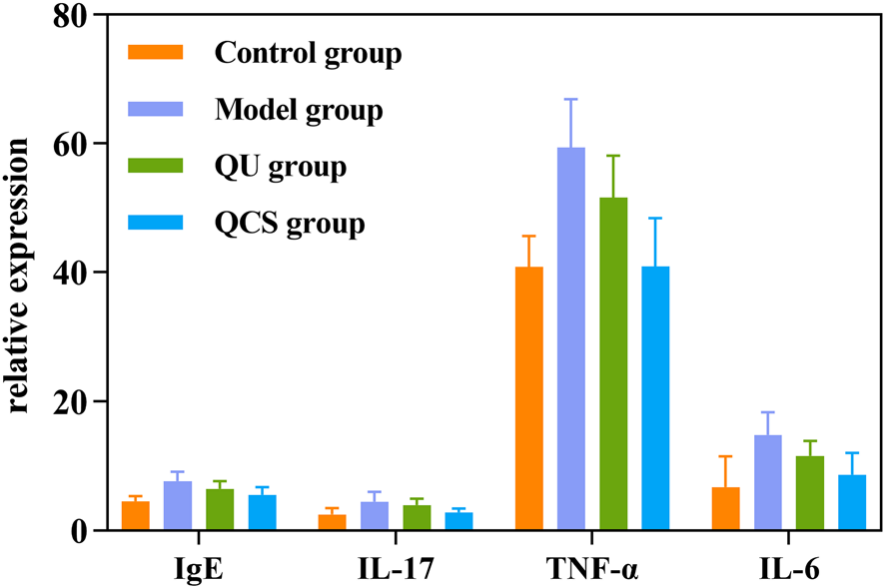
Comparison of expression of proinflammatory factors among the four groups.

As shown in Fig. 7, the highest expression levels of all factors were observed in the model group, followed by the QU group, while the levels in the QCS group approached those in the control group. After the induction of nasal mucosal inflammation, the changes in IgE, IL-17, TNF-α, and IL-6 concentrations in the model group indicated upregulation of these markers and suggested successful induction of inflammation. Treatment with QU alone did not lead to a significant decrease in levels of these markers, which indicated that its effectiveness in controlling inflammation was limited. However, after treatment with QU loaded on a CS nanoplatform, the expression of these markers significantly decreased and even approached the levels in the control group. This demonstrated the excellent downregulating effect of QCS on the proinflammatory factors and its superior efficacy in controlling inflammation in AR.

#### Histopathological analysis

The nasal cavities of mice were excised and fixed in 4% paraformaldehyde at room temperature prior to decalcification. After decalcification, the nasal tissues were embedded in paraffin and cut into 3 μm cross-sections. Subsequently, the sections were stained with H&E to assess eosinophil infiltration. Three independent observers, who were blinded to the experimental conditions, counted the numbers of eosinophils and mast cells infiltrating the nasal mucosa in five randomly selected sections using high-power fields under an optical microscope, as shown in Table 5. The averages of the counts from the three observers were used for statistical analysis.

**Table 5:** Quantitative scoring criteria for nasal mucosal pathology in mice.

Symptom	0 points	1 point	2 points	3 points
Degree of infiltration by inflammatory cells	No inflammatory cells observed	A few scattered inflammatory cells	A significant number of inflammatory cells	Occupying more than two-thirds of the entire epithelium
Degree of epithelial metaplasia	No epithelial metaplasia observed	Occupying one-third of the entire epithelium	Occupying one-third to two-thirds of the entire epithelium	Occupying more than two-thirds of the entire epithelium
Increased proliferation of glandular tissue in the submucosa	No glandular tissue observed	Glandular tissue occupying less than one-third of the submucosa	Glandular tissue occupying one-third to two-thirds of the submucosa	Glandular tissue occupying more than two-thirds of the submucosa
Dilated and congested blood vessels	No abnormal blood vessels observed	Mild dilation and congestion of blood vessels	Significant dilation and congestion of blood vessels	Combined with hyperplasia of blood vessels

At the end of the study, three mice were randomly selected from each group for anesthesia and euthanasia. The nasal mucosa was then collected, fixed in 40 ng L^−1^ polyformaldehyde for 48 h, dehydrated, embedded, and sliced to a thickness of 3 μm. The sections were stained with H&E and examined under an optical microscope to observe pathological changes in the nasal mucosa. Ten representative fields at a magnification of 400× were chosen to assess the pathological conditions of the mucosal epithelium, lamina propria, basement membrane, and submucosal layer, and a scoring system based on literature standards^20^ was used for quantification.

Observations revealed that the nasal mucosa of the control group mice was covered with transitional and pseudostratified columnar epithelium with a uniform and normal cell arrangement. In the model group mice, the nasal mucosal tissue exhibited a disorganized structure, interstitial edema, extensive exfoliation and metaplasia of the epithelium, and significant infiltration of inflammatory cells. In comparison with the model group, the mice in the QU group displayed less infiltration of inflammatory cells in the nasal mucosa, and slight regeneration of microvilli was observed in the epithelial layer. In the QCS group, the structure of the nasal mucosa appeared almost indistinguishable from that in the control group, with no interstitial edema. The integrity of the mucosal epithelium was further restored, with no significant loss of ciliated epithelium and an organized orientation of cilia.

**Figure 8:**
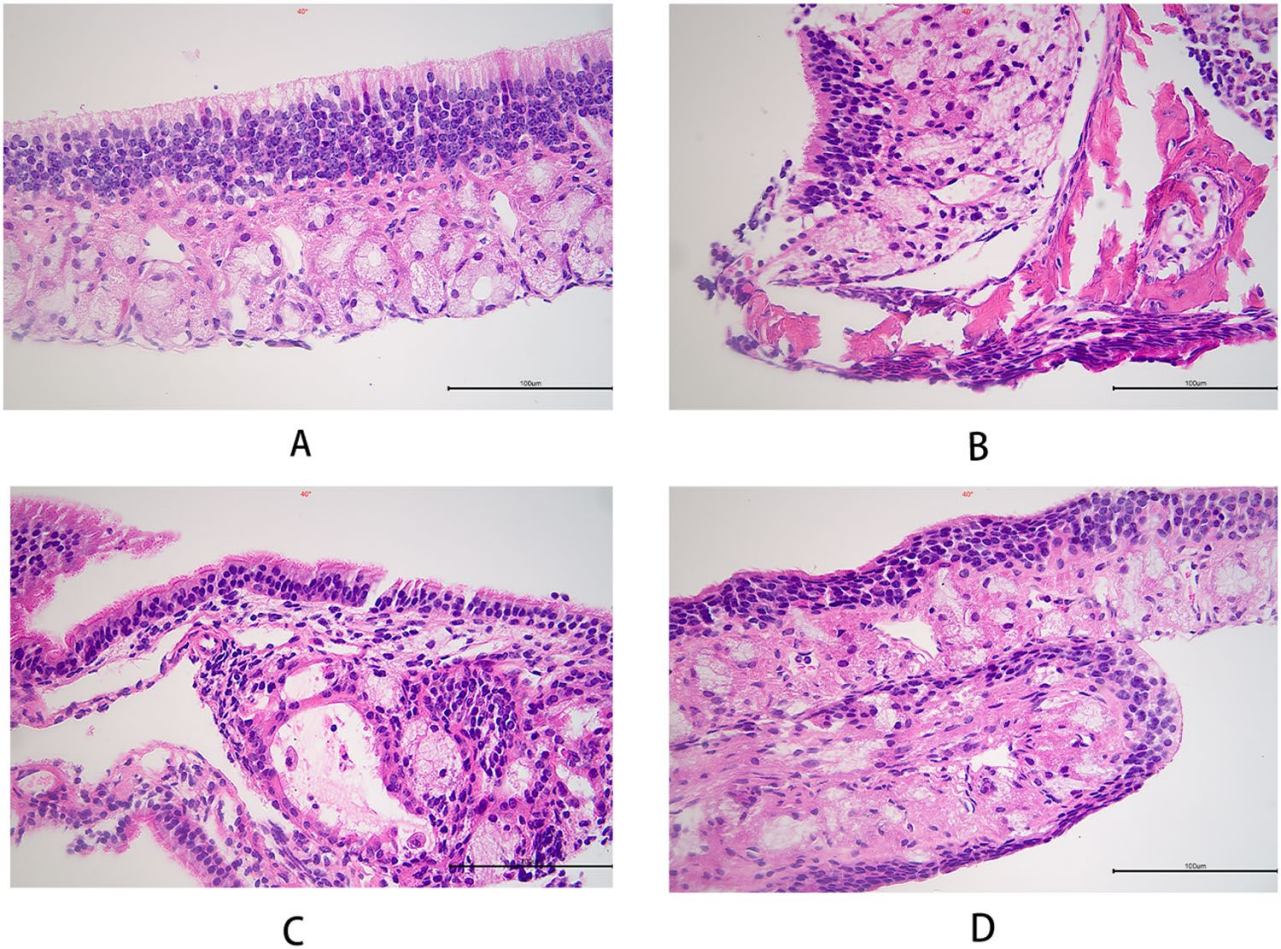
Hematoxylin and eosin staining of nasal mucosa from mice in the four groups. (**A**) Control group; (**B**) model group; (**C**) QU group; (**D**) QCS group. Magnification: ×400.

Data analysis using the scoring system showed a statistically significant increase in the nasal mucosal pathology score in the model group in comparison with the control group (*p* < 0.05). In comparison with the model group, both the QU group and the QCS group exhibited a statistically significant decrease in the nasal mucosal pathology score (*p* < 0.05). In addition, when the QCS group was compared with the QU group, a statistically significant decrease in the nasal mucosal pathology score was observed (*p* < 0.05). Figure 8 and Table 6 provide more details.

**Table 6:** Comparison of nasal mucosal pathology scores in the different groups (mean ± SD).

Group	Score after administration for 14 days (n = 3)
Control group	3.13 ± 0.35
Model group	9.33 ± 0.89*
QU group	7.96 ± 0.46#
QCS group	5.67 ± 0.47&

*p < 0.05 in comparison with the control group; ^#^*p* < 0.05 in comparison with the model group; ^&^*p* < 0.05 in comparison with the QU group.

## Discussion

In this study, a biodegradable topical nanoplatform based on CS was developed for the targeted release of QU, which is a pH-sensitive compound, to the nasal mucosa for the treatment of AR. The stability and functionality of nanocarriers and nanoparticles depend on their physicochemical properties, such as zeta-potential, shape, average size, and PDI. Literature research has shown that CS nanoparticles exhibit high stability as drug carriers and there is a strong ionic interaction with QU encapsulated within CS nanocapsules^21^. During the drug testing process, we found that the combination of ionic crosslinking and the excellent biocompatibility of CS effectively enhanced the binding between QU and CS, which resulted in favorable particle sizes, binding stability, and PDI. The binding of QU with CS improved its release efficiency and dissolution rate in the slightly acidic environment of the nasal cavity, which led to enhanced sensitivity overall. The encapsulation efficiency, drug loading capacity, and drug release levels significantly improved, and no apparent side effects were observed, which is consistent with our previous studies of QU and CS.

In animal experiments, IgE is selected as one of the indicators to investigate the occurrence of AR and the process of treatment. Moreover, changes in the Th1/Th2 pathway and Th17/regulatory T cell (Treg) pathway in AR are studied to elucidate the mechanism of immune imbalance involved. Th1 cell-mediated secretion of TNF-α, Th2 cell-mediated secretion of IL-6, and IL-17 within the Th17/Treg pathway are chosen as indicators for investigation.

IgE plays a crucial role in the occurrence and continuance of allergic diseases, and various forms of IgE-related allergic diseases affect approximately 30% of the world's population. The symptoms range from relatively mild conditions, such as rhinitis, to potentially life-threatening conditions, such as asthma or allergic reactions ^22^. During the sensitization phase, upon re-exposure of the airway wall to the sensitizing agent, memory B cells can be directly stimulated to differentiate into plasma cells and secrete a large amount of IgE antibodies. These IgE antibodies bind to high-affinity FcεRI receptors on the surface of mast cells, and, when the sensitizing agent binds to IgE on mast cells, a “bridging” reaction occurs, which triggers degranulation of cellular vesicles. Subsequently, mast cells release a series of preformed and newly synthesized proinflammatory mediators.

The release of histamine and proteases by mast cells during the effector phase of allergic reactions triggers local inflammation, which leads to symptoms such as watery nasal discharge, sneezing, and nasal itching. This process, which is known as the immediate hypersensitivity reaction, occurs within seconds to minutes after stimulation and generally subsides after a few hours. Chemotactic cytokines collaboratively participate in this process, which results in infiltration of immune cells into nasal mucosal tissues. Once activated, the infiltrated inflammatory cells release a large amount of proinflammatory mediators and cytokines, such as IL-5, IL-13, IL-17, IL-31, and IL-33, which collectively contribute to the development of strong inflammatory reactions and thus trigger recurrent episodes of acute allergic symptoms^23^.

The pathogenesis of AR also involves the participation of Th1, Th17, and Treg cells and macrophages. When the body is exposed to abnormal antigenic stimulation, the balance between differentiation of Th1 and Th2 cells is disrupted, with a weakened Th1 cell response and an enhanced Th2 cell response, which leads to dysregulated immune responses ^24^.

CD4^+^ Th cells are the principal cells in the immune system. Initially, naïve T cells are activated and differentiate into various functional subsets, including Th1, Th2, and Th17 cells, upon antigenic stimulation^25^. Th1 cells secrete IL-2, IL-8, IFN-γ, TNF-α, and other cytokines, which mediate cellular immune responses and organ-specific autoimmune diseases. Th2 cells produce IL-4, IL-6, IL-10, and other factors that primarily mediate humoral immune responses. It has been observed that Th1 cells mainly secrete proinflammatory cytokines, while Th2 cells mainly secrete anti-inflammatory cytokines to maintain normal immune function^26^. TNF-α is pleiotropic multifunctional cytokine mainly secreted by activated macrophages, natural killer cells, and T cells. It is involved in immune response, anti-infection, local inflammation, and endothelial cell activation processes.

IL-6 can be secreted by various types of cells, such as activated T cells, B cells, fibroblasts, monocytes, and macrophages. It is a multifunctional cytokine that can regulate the immune responses of the body, participate in the acute-phase reaction, and play an important role in the body's immune response to infections ^27^. IL-6 can regulate the immune responses of the body by mediating neutrophils and macrophages. It can also promote the differentiation of T and B cells and thereby enhance the specificity of the immune response against infected cells and cells with characteristics of specific foreign antigens^28^. Related research ^29^ has shown that *IL6* and *TNF* are two pivotal genes associated with the toll-like receptor signaling pathway. IL-6 and TNF-α are important proinflammatory cytokines that are frequently found in various parts of the body during inflammatory reactions. They enhance inflammatory responses and have been shown to be associated with AR ^30^. They are secreted by Th2 cells and Th1 cells, respectively, and are involved in regulation of the Th1/Th2 balance. Therefore, these two indicators are selected as markers for evaluation.

Th17 cells have been shown to be associated with the generation of Th2 cells and can promote goblet cell hyperplasia and the production of mucin in allergic diseases ^31^. They maintain a balance with Treg cells, with which they share common precursor cells (naïve CD4^+^ T cells) and require a common tumor growth factor-β signal for initial differentiation. However, the terminally differentiated cells exhibit opposing functions: Th17 cells induce autoimmunity and inflammation, while Treg cells suppress these phenomena and maintain immune homeostasis ^32^. Excessive activation of Th17 cells can lead to an excessive inflammatory response and even the development of autoimmune diseases^33^. The cytokines of the IL-17 family have been recognized over the past two decades as a group of molecules involved in various physiological or pathological processes of mucosal barrier tissues, and they exert either stimulatory or inhibitory effects^34^. Th17 cells are the main producers of IL-17, in patients with AR caused by pollen allergens, the serum level of IL-17 significantly increases during pollen seasons and displays a positive correlation with the severity of clinical symptoms. Modulating the balance between Treg and Th17 cells represents a novel approach in the treatment of inflammatory diseases. The significant changes in IL-17 concentrations observed in this study indicate that IL-17 is involved in the regulation of AR attacks in mice.

## Conclusions

According to our research, the use of QCS and its anti-inflammatory properties in the treatment of AR has shown significant therapeutic effects in animal experiments. Animal experiments have demonstrated that, in a mouse model of AR induced by ovalbumin, QCS, in comparison with QU alone, displayed significantly enhanced anti-inflammatory effects by decreasing the levels of IgE, IL-17, TNF-α, and IL-6. This indicates that it regulated and corrected Th1/Th2 and Th17/Treg imbalances during the development of AR. These research findings support the use of nasal nanodrug formulations combining QU and CS as a new pharmaceutical approach that may play a positive role in the long-term treatment of AR without adverse effects. However, the understanding of the mechanism of action of QCS is still limited. Further investigations are therefore required in the form of cell experiments, as well as studies of practical aspects of drug administration, such as passing the mucosal barrier and enhancing the mucoadhesive properties of the drug in the nasal microenvironment, to achieve multi-responsive drug release for faster therapeutic effects. This requires further research and improvement to enhance the efficacy of this drug.

## Data Availability

The data presented in this study are available on request from the corresponding author.
